# Behaviour of *Vespa velutina nigrithorax* (Hymenoptera: Vespidae) under Controlled Environmental Conditions

**DOI:** 10.3390/insects14010059

**Published:** 2023-01-07

**Authors:** Omaira de la Hera, María Luz Alonso, Rosa María Alonso

**Affiliations:** Department of Analytical Chemistry, Faculty of Science and Technology, University of the Basque Country (UPV/EHU), Barrio Sarriena, s/n, 48940 Leioa, Spain

**Keywords:** *Vespa velutina nigrithorax*, behavioural study, embryo and secondary nest

## Abstract

**Simple Summary:**

*Vespa velutina*, commonly known as the Asian hornet, is an exotic species originating from the Asian continent. It has become an invasive species in Europe due to it being a predator of native fruits and insects, especially honeybees. The aim of this work was to carry out an observational study of the *Vespa velutina* behaviour in captivity. Two secondary and one embryo nests were kept under controlled environmental conditions. Captivity adaptation, defence against perturbations, evolution of the colony and overwintering were the different behaviours studied. This work has shown, for the first time up to our knowledge, the possibility to maintain secondary nests up to 13 weeks under controlled environmental conditions. The embryo nest was kept up to 6 weeks in captivity. The research carried out in this work has allowed deepening our knowledge of *Vespa velutina*’s ethology, obtaining useful information on the behaviour of this invasive species.

**Abstract:**

From its introduction in Europe, *Vespa velutina nigrithorax* has become an invasive species, since it is a predator of native fruits and insects, most of the latter being honeybees. Despite the knowledge on the life cycle of this hornet, Asian hornet behaviour is not well understood, since in vivo studies on this species are quite difficult to perform. In this work, an observational study of the behaviour of this invasive species in captivity has been carried out. Two secondary and one embryo nests were caught and kept under controlled environmental conditions, up to 13 weeks for the secondary nest and 6 weeks for the embryo nest. Captivity adaptation, defence against perturbations, evolution of the colony and overwintering were the different behaviours studied. The study has shown the importance of avoiding disturbances to the nest from the beginning of the experiments, since they tend to destroy the colony. The aggressive behaviour observed in the embryo nest was lower than in the secondary nests. Results of this research will allow obtaining additional information on this species, which is crucial to develop effective control methods.

## 1. Introduction

Biological invasions have increased due to globalization and the connections generated by world commerce. In this sense, invasive species represent a risk for diversity [1,2]. The ecological and economic problems produced by biological invasions have led to them being considered as a major concern all over the world. Social insects such as social wasps and hornets are particularly efficient invaders [2] and have different degrees of sociability. The eusocial insects show one of the most developed social behaviours, and include some species of bees, wasp, ants, termites, or hornets, such as *Vespa velutina* (Lepeletier, 1836).

*Vespa velutina*, commonly known as the Asian hornet, is an exotic species originating from Asian continent. In 2004, an overwintering queen was accidentally introduced to France from China, and within a few years it was spread throughout the west of Europe [3,4,5]. The presence of Asian hornets outside their natural habitat has caused a great environmental threat because it is a predator of native fruits and insects, most of the latter being honeybees (*Apis mellifera*, Linnaeus, 1758) [6,7,8,9,10,11].

These hornets, like other eusocial insects, live in large colonies, which are usually monospecific, consisting of hundreds to thousands of individuals, which according to their task are divided in castes [12]. The queen is responsible of building the embryo nest and laying eggs; males are only engaged in mating with the future queens and workers are responsible of the colony growth by building and repairing the nest, defending it, and feeding larvae. The broods are fed with various kinds of insects and arachnids, which provide them protein, while the adult diet is based on carbohydrates obtained from fruits or flower nectar [13].

**Figure 1:**
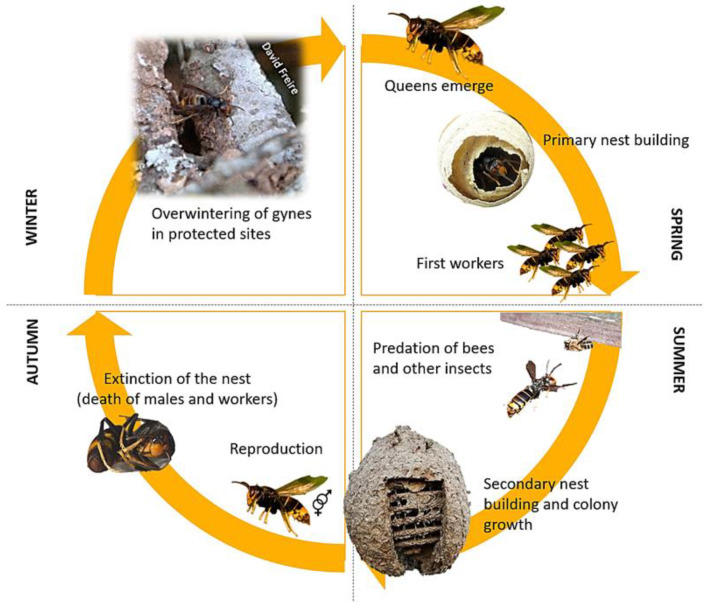
Life cycle of *Vespa velutina* hornet.

Despite the knowledge on the life cycle of this hornet (Figure 1), nowadays, Asian hornet behaviour is not well understood, since in vivo studies on this species are quite difficult to perform. The reasons for this is that the majority of the colonies builds their nests at the top of the leafy trees, making their visibility and access for humans difficult, until leaves fall. It is well known that they have a short life cycle [14]. A single queen produces an annual colony [11] and if the nest is disturbed within a 10 m distance, they defend it aggressively [15,16], which makes it very difficult to obtain data on colony behaviour [17]. Apart from this, there is threat to take or study the nest colony without protection due to its painful and dangerous sting [5].

Very few works are found in bibliography focused on the behavioural study of *Vespa velutina* in captivity. Nest defence, the colony activity and olfactory attraction are the behavioural studies reported.

Its defensive behaviour as a function of age in captive hornets was studied by Monceau et al. [13]. They found a relationship between worker age and nest defence, where the oldest hornets were the first to expose themselves to danger with the aim of defending the colony.

Monceu et al. [13] and Perrard et al. [18] carried out observations of the hornet’s activity and foraging behaviour of an embryo nest in captivity in an outdoor cage. An increase in both nest size (up to 38 cm) and colony activity when the nest is in semi-captivity was detected. However, authors also observed a significant difference in the size between nests in the wild and in captivity, the former being larger.

Furthermore, *Vespa velutina*’s olfactory attraction was studied by Couto et al. [2] and Monceau et al. [13]. With this purpose, they put the hornets in contact with different attractants, such as meat and fish protein, honey, pollen, honeycomb wax, propolis, queen bee pheromones, worker bees, and bee larvae. Hive products and pheromones emitted by the bees were found as the most attractive food for *Vespa velutina*.

Moreover, as Chapuisant and Keller [19] reported, the study of social insects in captivity allows quantifying the longevity in the absence of external mortality pressures. This makes it easier to carry out research in different areas, such as genetic studies, identification of chemical compounds, attractant–repellent tests, behavioural studies, etc., since research groups will manage to have available hornets, whole nests or larvae during long periods.

Conclusions extracted from the behavioural studies are crucial to develop effective control methods for this invasive species. Up to now, attractive traps, the introduction of biocides inside the nest by means of different tools such as poles, drones and paintball guns, and protein baits, among others, are the current methods used [20,21].

Therefore, the aim of this work was to carry out an observational study of the behaviour of *Vespa velutina* in captivity. For that purpose, two secondary and one embryo nests were caught and kept under controlled environmental conditions up to 13 weeks for the secondary nest and 6 weeks for the embryo nest. Captivity adaptation, defence against perturbations, evolution of the colony and overwintering were the different behaviours studied. Results of this research will allow us to obtain additional information on this invasive species. This opens the door to obtain larvae and hornets out their natural seasonal period of their life cycle, in order to perform different research studies.

## 2. Materials and Methods

### 2.1. Nest Collection

On 19 November 2019 and 13 March 2020 two secondary nests were supplied by Basalan (Biscay Provincial Council, Lezama, Spain) and the Avispa Asiatica Association (Burgos, Villarcayo, Spain), respectively. The first nest was located on the roof of a garage about 3 m above the ground in Mungia, Bizkaia, Spain, and the second was inside a bush at ground level in Hernani, Gipuzkoa, Spain.

An embryo nest was collected 17 May 2022 in Llodio, Araba, Spain. This was collected above the window of a house, by Araba Firefighters.

**Figure 2:**
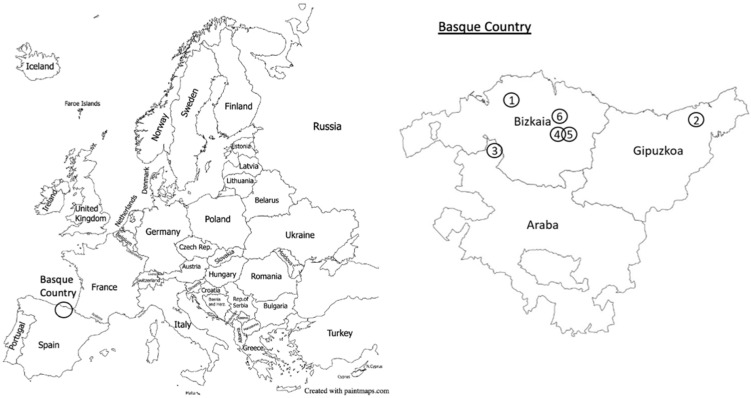
Location of the Basque Country in Europe (**left**). Map of the Basque Country, with its three counties (**right**). The numbers indicate the locations of the *Vespa velutina* supplied nests. Image modified from paintmaps.com (accessed on 4 September 2022).

In addition to these nests, on 24 and 27 September and 1 October 2021, three secondary nests were supplied, which were used for experiments of other research projects and to corroborate the defensive behaviour of hornets from the collection to the arrival at the laboratory. The three nests were supplied by Basalan. The two first nests were collected in Amorebieta, Bizkaia, Spain and the last one in Ajangiz, Bizkaia, Spain. In Table 1 and Figure 2, the locations of the different nest are given.

For the collection and handling of the different nests, a certified protective suit from Xorsa workwear (Xorsa Global, Lugo, Spain), consisting of a coverall with an adrenaline pocket on the left arm, protective hood for the head, and gloves and boot covers, was used. The suit is certified by the Aitex Textile Technological Institute (Alcoy, Spain) (No. 0161/4401/15ar), under the EN ISO 13688:2013 standards of the National Accreditation Body (ENAC) for protective clothing.

The nests were collected in transparent plastic boxes, which had little holes to allow hornets’ breathing. The closure of the boxes was sealed with adhesive tape.

### 2.2. Handling of Vespa velutina Nests in the Laboratory

To handle *Vespa velutina* nests, the first step was to anaesthetise the hornets using diethyl ether (99.7%) from Panreac Applichem (Barcelona, Spain) as an anaesthetic.

For this purpose, the holes made in a transport box that allowed the nest to breathe were covered with adhesive tape, except one through which the tip of the syringe with anaesthetic was inserted. The amount of anaesthetic needed to sleep them depended on the size of the box and the nest, as well as the structure of the nest. The anaesthetic should be added little by little injecting each time a volume of about 2 mL, until all the hornets are asleep, since an excess could kill the hornets. Due to this fact, the nests should be transported in transparent boxes that allow a clear view inside.

Once hornets were asleep, before the captivity assay, the external dimensions were measured. Then, nests were placed in the cage along with all the sleeping hornets that were nesting outside. In the case of the embryo nest, it was hung on the upper part of the cage in the position in which it would be found in the wild.

### 2.3. Cage for Captivity Assays

The nests and hornets were placed in a cage with a wooden structure (1 × 1 × 1 m). A transparent methacrylate sheet stood at the front of the cage, allowing the interior view. In order to achieve good ventilation for the hornets, the other sides of the structure were covered with a fiberglass grid with 5 mm holes. In order to facilitate the introduction of the nest in one of the sides, the grid was attached to the cage with VELCRO^®^ (Velcro Europe, Barcelona, Spain). At the bottom, the cage had a methacrylate drawer, which facilitated cleaning and food placement. Furthermore, the cage had a locking system; when the drawer was removed, a trap door dropped and covered the hole in the cage, preventing the hornets from escaping.

**Figure 3:**
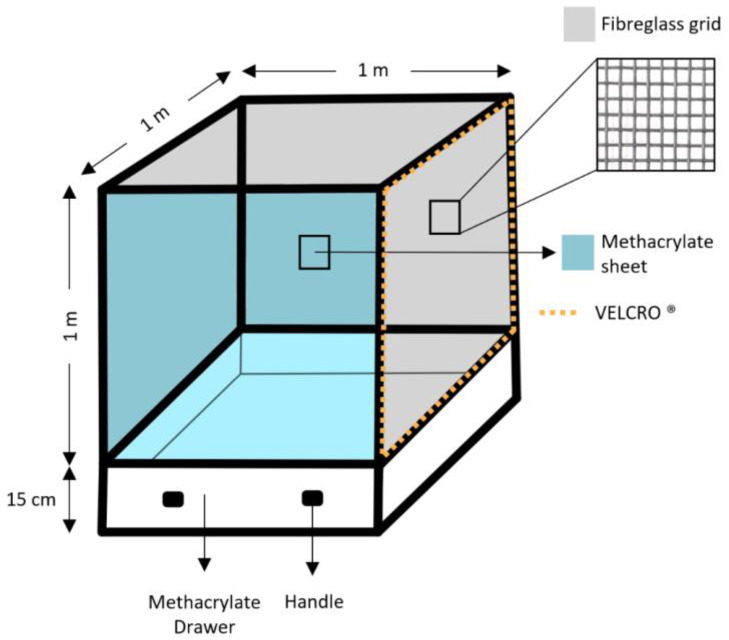
Scheme of the cage used for the captivity study of *Vespa velutina*.

The Figure 3 shows a scheme of the built cage used for the captivity studies.

### 2.4. Study of Vespa velutina Nest in Captivity

Every day, the hornets in the cage were fed with a mixture of honey–water (50:50) and different pieces of a variety of fruits (banana, grape and apple) [17,18,22], which were placed on the side grids and inside the cage. In addition, common cleaning paper impregnated with a mixture of water and honey was placed on the upper grid. Moreover, fish protein, which is the main component of the bait pieces tested, was provided. Water and honey–water mixture were given ad libitum as other studies reported [14,18,23]

For the captivity study the nests were adapted to a temperature-controlled environment (25–28 °C) with light cycles (12 h). In addition, they were provided with different materials that served as complementary food or to condition the nest (tree bark, fruit plants, stones and grass) [24]. Additionally, in the case of the embryo nest, pieces of *Vespa velutina* nests were added to facilitate the repair and growth of the nest.

The different behaviours observed in the hornets were recorded and annotated every morning and evening during at least one hour.

Observations were grouped according to captivity adaptation behaviour, defence against perturbations, the evolution of the colony and finally, overwintering. Once we finished the experiments, the number of individuals was counted, differentiating between males and females.

To study their defensive behaviour, apart from their response to disturbances, two tests were carried out. The first consisted of simulating an attack towards a group of hornets with a wooden stick, which was moved and waved near to them. The second was a blowing test, in which a current of air was send towards the hornets. These tests were applied to the hornets of both secondary nests.

For overwintering experiments, environmental conditions of the two secondary nests were changed. Food and water were removed, the temperature was decreased to 13–15 °C and the light was turned off.

It must be considered that animals living and growing up in captivity experience drastically different conditions to those of the species in the wild, and therefore may act differently [25].

## 3. Results and Discussion

The first secondary *Vespa velutina* nest studied was kept in captivity for 13 weeks from its reception in the laboratory, with 8 weeks in full activity and 5 weeks in a lethargic state. The second nest was maintained for 15 weeks in full activity.

The embryo nest collected in May was kept up to 6 weeks. Other authors performed captivity experiments with nests; Bonnard et al. [26], maintained a secondary nest for 4 weeks and two late embryo nests up to 41 days in captivity conditions. Furthermore, Perrard et al. [18] carried out studies to observe the activity of a colony for 20 weeks. The study was done with an embryo nest in a cage placed outside. In addition, the cage was closed and kept under controlled environmental conditions for only four weeks, but after this time until the end of the assay the cage was opened, allowing the hornets to go outside to forage.

However, Hoffmann et al. [23] were able to successfully record the complete life cycle of *Vespa crabro* (Linnaeus, 1758), the European counterpart to *Vespa velutina*, in captivity from two secondary nests during the mating season. This study allowed them to observe mating, hibernation, nest initiation, colony growth and the birth of males and new gynes.

Several authors have kept *Vespa velutina* in captivity for various behavioural assays such as the study of the relationship of hornet age with defensive behaviour [13], or the olfactory attraction of the species to different foods, as well as to attractant molecules [2]. Nevertheless, in these cases, the maintenance of the species over time was not the main objective. The different groups of behavioural observations carried out during the captivity studies in this work are next described.

### 3.1. Adaptation to Captivity Conditions

**Figure 4:**
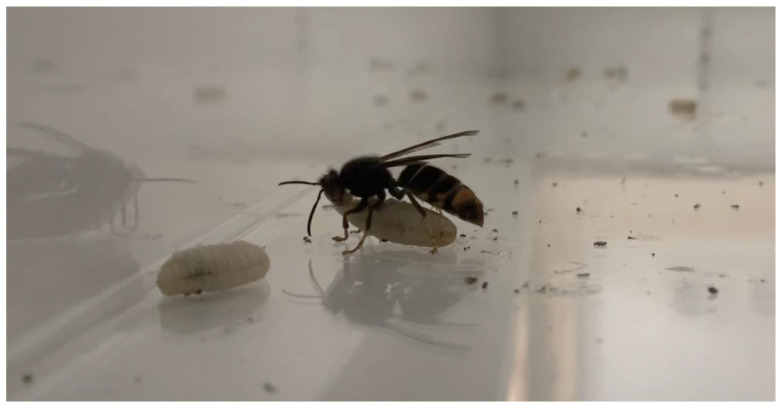
Adult *Vespa velutina* hornet removing a larva from the nest, with another previously removed larva.

During the first two days after they woke up, the hornets, in each nest, attacked and destroyed it, removed the larvae and transported them around the cage (Figure 4). The larvae were even found inside the drinker. Although this behaviour also occurred in the embryo nest, it was less pronounced. This may be due to, as Perrard et al. [18] suggested, the fact that embryo nests of *Vespa velutina* are less aggressive towards humans, and thus towards their own colony. In addition, it has been reported that colonies close to residential areas get used to the human’s presence.

It is important to note that this behaviour started to be displayed before the captivity experiment. Prior to being anaesthetised, the hornets in the transport boxes showed aggressive behaviour towards the outside and from the first minutes, the hornets removed the larvae from the nest cells.

**Figure 5:**
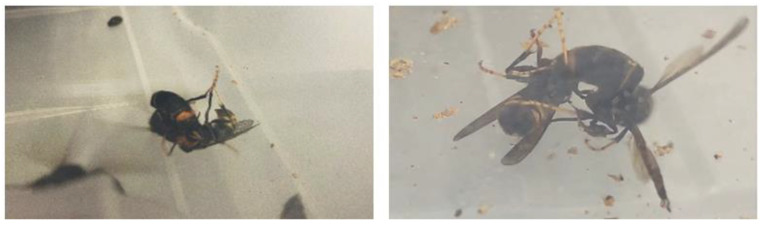
*Vespa velutina* female hornets fighting each other shortly after the nest was collected.

From the observation of these aggressive behaviours, it can be concluded that the *Vespa velutina* species, in a state of threat or stress, becomes more aggressive and attacks each other (Figure 5).

**Figure 6:**
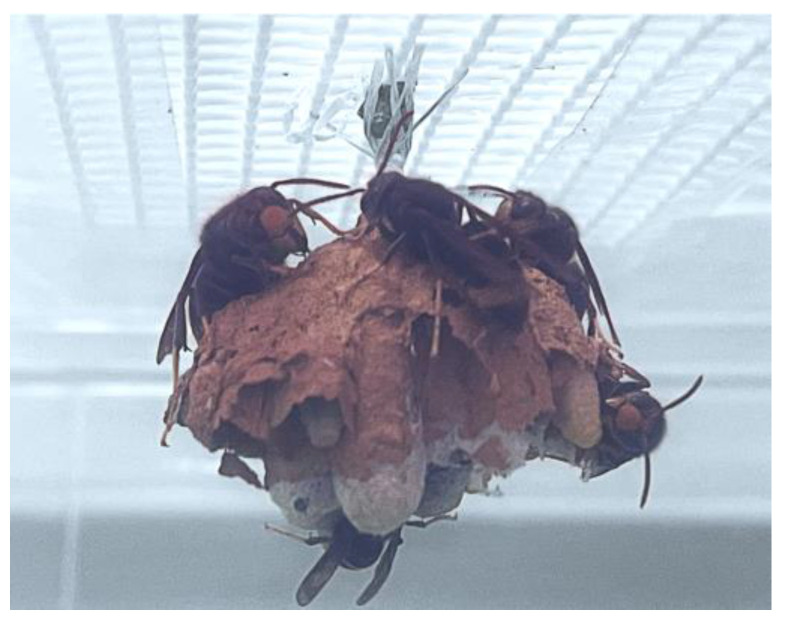
Embryo nest of *Vespa velutina* in the captivity cage after 2 days of adaptation.

Regarding the embryo nest, the hornets’ aggressive behaviour was also observed, since they destroyed the external cover of the nest, leaving the cells uncovered. This made it possible to visualise the colony from inside the nest from the beginning of the captivity experiment (Figure 6).

Regarding their behaviour with respect to the intake of food, one day after the reception of the nest and once all the hornets woke up, they showed a pretty nervous behaviour. In fact, when food and water were provided, they ignored it, and water was thrown out of the drinker.

This nervousness is not something new, since the study done by Perrard et al. [18] on the observation of colony activity kept in an outside cage demonstrated that handling the nest caused a disturbance during the first 2 days of captivity.

After approximately two days, this behaviour stopped, they were more relaxed, and began to take the food and drink water.

**Figure 7:**
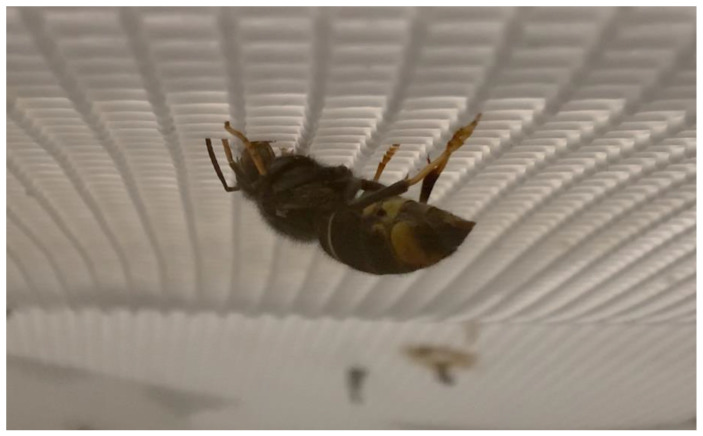
*Vespa velutina* hornet taking food from the impregnated paper on the ceiling.

In particular, initially, the majority of hornets only took food from the second day onwards. This conduct could be observed since they started after 3 days to take the food and the water–honey mixture impregnated in the paper, which was placed on the walls and ceiling of the cage (Figure 7).

It was observed that the hornets associated the laying of new food with the removal of the dry impregnated paper, as they instantly moved without aggression to the ceiling of the cage.

With respect to the protein food given to the hornets, they did not take it, ignoring it. This behaviour could be due to the lack of live larvae inside the nest or because they were affected by being outside their natural habitat. The most reliable hypothesis is the lack of larvae, due to the absence of scratching noises from inside the nest, which are characteristic noises from larvae asking food [18].

Due to the lack of live larvae, it was not possible to obtain information on what happened in the nest when bait rich in protein was introduced.

Nevertheless, in the embryo nest the survival of the larvae and how they were fed by the workers could be verified. In this nest, workers were seen taking protein food to feed the larvae. These results open the door to create in vivo studies using embryo nests to evaluate the efficacy of protein baits to fight against this invasive species.

### 3.2. Defence against Perturbations

The hornets showed a defensive attitude when disturbed. On the one hand, as has been described previously in the adaptation section, the aggressive behaviour during the first days of captivity is a defensive behaviour towards external perturbations. On the other hand, after two days of adaptations, the hornets reacted against perturbations as when the cage was moved during feeding, cleaning or when the drinker was removed. The hornets inside quickly left the nest; some of them stayed near the nest entrance, while the others moved aggressively towards the affected zone, defending the colony.

The response of hornets to the two tests applied was the following: regarding the wooden stick test, they responded by stinging and biting it. Concerning the blowing test, hornets flew aggressively into the air stream and once there, they bitted the plastic grid.

These reactions were expected and well known, as they were described in the research carried out by Monceau et al. [13], where they studied the relationship between the age of *Vespa velutina* workers and the defensive behaviour. When simulating attacks on the nest with large tweezers, they observed aggressive behaviour. As reaction, hornets attacked the tweezers with bites and stinging.

### 3.3. Evolution of the Colony

Once the hornets were relaxed and adapted to the cage, the evolution of the colony in captivity under the before mentioned conditions was studied.

**Figure 8:**
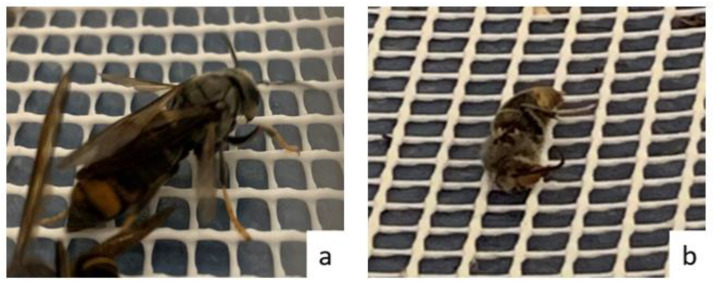
Newly born in captivity *Vespa velutina* with the characteristic whitish colouring of the first days after hatching (**a**). Attacked male *Vespa velutina* hornet with some of its legs and wings lost (**b**).

During the first two weeks, new individuals were born, mostly males, which hatched from pupae already at the nest reception. These were identified by their characteristic whitish coloration in the first days after hatching, due to the lack of keratin (Figure 8a). However, in the embryo nest, as is expected in this period of the life cycle, the majority of newborns were females.

However, the number of new hornets decreased throughout the captive experiments. Monceau et al. [13] observed the same evolution in their research. In particular, that study suggested that the queen stopped producing eggs after caging. The stress of captivity, lack of nutrients or resources needed to regulate the colony or its health maintenance could be the hypothesis for the failure of the queen not laying eggs under captivity, and hence result in the halt in the colony growth.

Another aspect noted was the separation of genders, as occurs in the wild. As the new male individuals matured sexually, the females kept on or around the nest, while the males were in a corner of the cage grouped together. This fact was not perceived in the embryo nest, due to only three males being hatched, of which two were dead the day after hatching, and the last one failed to emerge from the pupa and died inside it.

In particular, in Nest 1, several female hornets were observed to be acting aggressively towards the male hornets, even attacking them with bites and stings until death. After the attacks, dismembered male hornets were seen (Figure 8b). This antagonistic behaviour, which was observed previously in *Vespa simillima* (Cameron 1903) might be a mechanism for workers to avoid inbreeding [13].

This behaviour between genders was also described in experiments carried out by Monceau et al. [13]. In their study, they observed that males occupied the walls of the cage. In addition, the workers showed aggressive behaviour, stinging and biting the males. After these attacks, they also observed sections of hornets in the cage.

**Figure 9:**
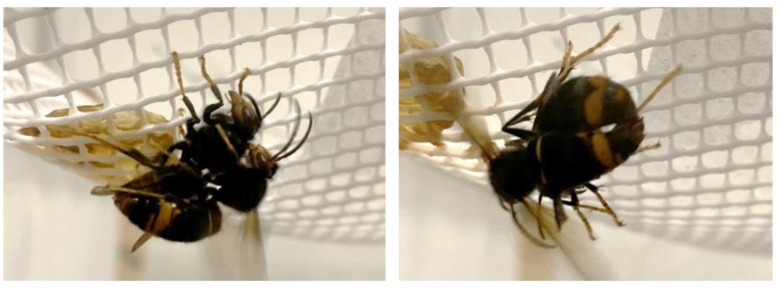
A couple of *Vespa velutina* from Nest 1 mating in captivity.

Since week 4 to 6, mating between hornets was detected on up to five times (Figure 9), in Nest 1. This act, as in the wild, took place in the areas of the cage furthest away from the nest. Therefore, mating in this nest took place without the need of mating areas as reported in the literature.

However, neither mating nor aggressive behaviour towards males was observed in Nest 2. These differences with respect to the first nest can be attributed to the fact that this nest was unusual, since it was collected in March, when there should not be any secondary nests. Therefore, the main hypothesis is that this nest has survived the winter, and the emerged gynes would have left the nest already fertilised, leaving only workers and males. This hypothesis can be endorsed by the possibility of the over-winter survival of a secondary nest described in the bibliography [27].

As the weeks went by, the number of individuals in both nests started to decrease, with us appreciating more dead hornets every day. This occurred from week 7 and 10 for Nest 1 and 2, respectively. Therefore, when the observed number of hornets in the cage was lower than 15, it was decided to decrease the temperature (from 25–28 °C to 16–17 °C), and food was gradually reduced until not provided, in order to simulate the arrival of autumn, when the hornets begin to prepare to go into a state of lethargy.

In the embryo nest, it was in week 5 when several hornets and larvae were found to be dead. In addition, the remaining larvae were less active than in previous weeks. It is remarkable that the queen, who had not left the nest before, was observed to be flying in the cage and taking food by herself.

The decrease in the number of workers could give rise to the larvae and the queen not receiving the necessary food, so that the queen had to feed and drink water by her own.

The following week the queen and the remaining workers and larvae were found dead.

### 3.4. Overwintering

**Figure 10:**
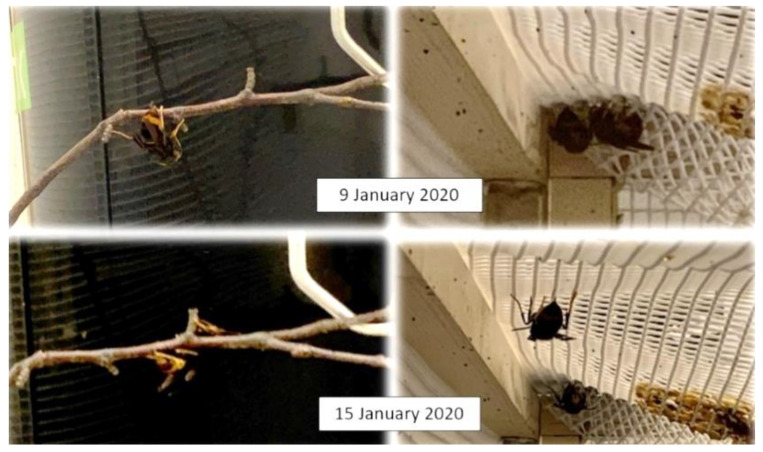
*Vespa velutina* hornets from nest 1 in state of lethargy in captivity.

After several days in unfed conditions, low temperature and without light cycles, in Nest 1, three large female hornets, possibly gynes, were found, one on a branch and the other two in a corner of the box without moving, possibly because they were in a state of lethargy. In order not to interfere with overwintering, their position was checked only once a week. As can be seen in Figure 10, after one week, the hornet on the branch maintained its position, while the one on the corner moved slightly from its initial position.

After 5 weeks of hibernation, due to technical problems in the forensic entomology laboratory that caused a failure in temperature and light conditions, the hornets awakened from overwintering. Therefore, it was decided to start a new biological cycle, so the environmental conditions were changed. The temperature was raised (21 °C), and the light cycles, as well as food and water, were reintroduced. The hornets returned to activity, ate, and flew normally. However, after a week, the three hornets were found dead. It is unknown why this happened, but it could be due to several factors such as the environment, which was not ideal to start the cycle again, or the existence of other gynes in the same environment, since in nature, for survival reasons, they do not hibernate in groups and individually build the primary nest away from other gynes.

**Figure 11:**
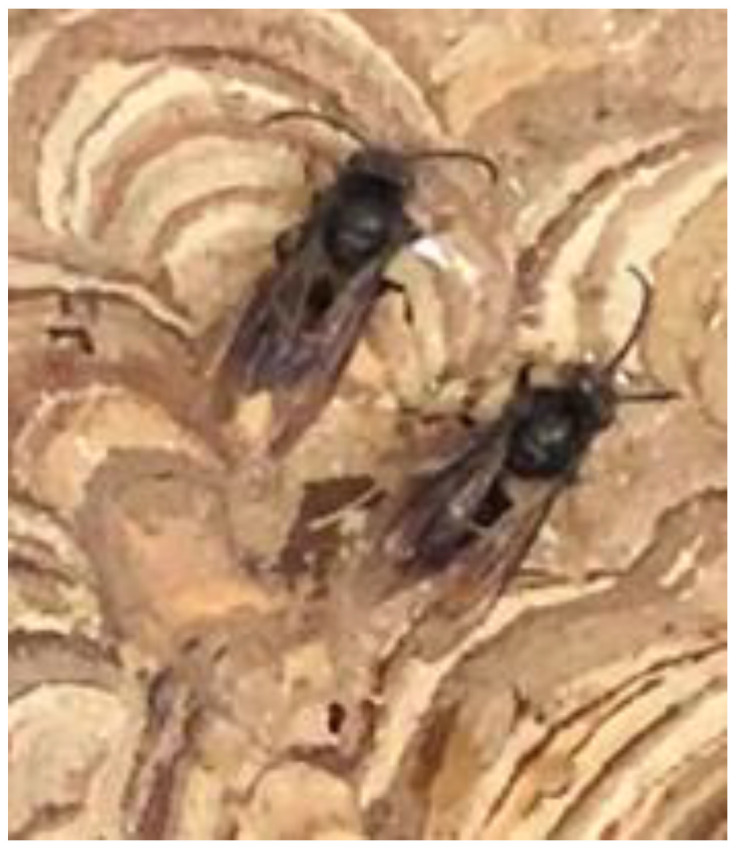
*Vespa velutina* male hornets in the nest.

Nevertheless, in Nest 2 after two weeks of non-feeding, there were 13 live male hornets. One week later there were no hornets left alive. The absence of gynes was suspected to be consequential to the death of the colony because only male hornets, as can be seen by the shape and length of their antennae [5], were observed in the nest (Figure 11).

### 3.5. Nest Structure and Composition after the Death of Colony

After the death of all hornets in the secondary nests, they were opened and the number of individuals was counted, differentiating between females and males.

In the case of the embryo nest, the colony composition from the beginning of the captivity experiment was known. It consisted of larvae (10 live and 8 operculated), six eggs, four workers and the queen.

As can be seen in Table 2, the number of males is higher than the number of females in the Nests 1 and 2. This agrees with Villemant et al. [28] and Monceau et al. [10], who pointed out that from mid-September the number of males is three times higher than the number of females, due to the fact that this is the mating season.

**Figure 12:**
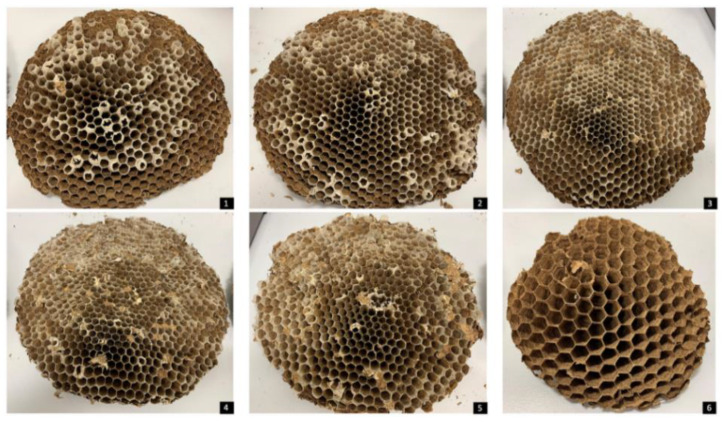
Combs from the first nest organized in order of formation: 1: first, 2: second, 3: third, 4: fourth, 5: fifth and 6: the last comb formed. (From upper to bottom).

It is important to note that Nests 1 and 2 showed the last comb, the last one built, with unused cells (Figure 12). This could verify the absence of new individuals after the hatching of all hornets that were operculated at the time of nest collection. However, this fact did not confirm the absence of the queen.

Dissection of the nests also revealed operculated larvae that failed to hatch, new hornets that were unable to emerge from the nest and dead larvae. The reasons for the failure of the operculated hornets to hatch are unknown. It should be noted that, as it was described by Perrard et al. [18], precise information on the structure and dynamics carried out in the colony is not available, as it was not possible to make observations inside the nest. Therefore, in future work it would be of special interest to incorporate cameras inside the nest to study the behaviour of the colony in its interior.

**Figure 13:**
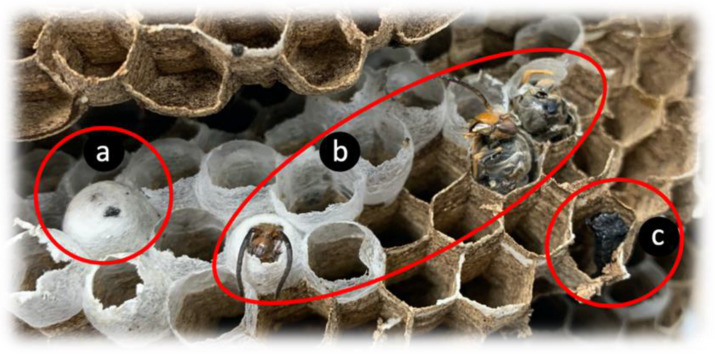
Nest individuals found after captivity studies. Operculated larvae that did not hatch (**a**), new hornets which did not emerge from the nest (**b**) and dead larvae (**c**).

The lack of nutrients or lack of feeding by the workers are some of the hypotheses to explain why the larvae did not survive and did not start pupation. Figure 13 shows the three types of individuals found inside the nest.

## 4. Conclusions

The observational study of *Vespa velutina* behaviour in captivity carried out has showed, for the first time up to our knowledge, the possibility to maintain secondary nests up to 13 weeks under controlled environmental conditions.

In this work, mating was observed in the secondary nest, despite the controlled environmental conditions, a similar behaviour as in wildlife. Therefore, it can be concluded that the life cycle of these hornets was completed from the stage of the nest at the time of collection.

The research carried out in this work has allowed deepening the knowledge surrounding *Vespa velutina’s* ethology, obtaining useful information on the behaviour of this invasive species.

## Figures and Tables

**Table 1:** Collection dates, locations, nest dimensions (total length, L, and width, W), and suppliers of the different nests of *Vespa velutina* for captivity and larvae studies.

Nest	Collection Date	Location	Dimensions (L × W)	Supplier
Captivity studies				
Secondary nests				
1	19 November 2019	Mungia (Bizkaia, Spain)	26 cm × 28 cm	Basalan
2	13 March 2020	Hernani (Gipuzkoa, Spain)	32 cm × 29 cm	Avispa Asiática Association
Embryo nest				
3	17 May 2022	Llodio (Araba, Spain)	5 cm × 5 cm	Araba Firefighters
Defensive behaviour				
Secondary nest				
4	24 September 2021	Amorebieta (Bizkaia, Spain)	42 cm × 41 cm	Basalan
5	27 September 2021		50 cm × 44 cm	
6	1 October 2021	Ajangiz (Bizkaia, Spain)	40 cm × 38 cm	

**Table 2:** Number of hornets counted in each nest after captive breeding in the laboratory.

Nest	1	2	3
Type of Nest	Secondary		Embryo
Captive time (weeks)	13	15	6
Number of Hornets			
Males	95	185	3
Females	76	125	10

## Data Availability

Data is contained within the article.
